# Twenty-two years of injury free

**DOI:** 10.1186/s40621-018-0137-z

**Published:** 2018-04-10

**Authors:** Lois K. Lee

**Affiliations:** Division of Emergency Medicine, Boston Children’s Hospital, Boston, MA 02115 USA

One of the extraordinary things about children is their constantly changing development and acquisition of skills. It is amazing to watch an infant who can roll, grow into a toddler who can run, then become a child who can ride a bicycle, and finally a teen who can drive a car. With this development and growth, also come different risks for varying mechanisms of injury throughout childhood and adolescence. Understanding these changing risks at different ages is important in trying to prevent injuries over their lifetime as injuries continue to be a leading cause of death and disability in pediatrics.

In this inaugural issue for the Injury Free Coalition for Kids® annual meeting supplement in Injury Epidemiology we have a range of studies addressing injury epidemiology and injury prevention initiatives that cover infants through the teen years. In the area of infant safe sleep, one study demonstrates safe sleep behaviors are compatible with mothers who breastfeed and another study highlights how specific messaging around safe sleep focused on fathers is also important. In this issue we also have several studies demonstrating how toddlers have varying risks for injuries to different parts of the body from falls. Younger toddlers have an increased risk for lower extremity injury with falls from slides, but have higher risks for head injuries for falls of other types, including from buildings. For teens, motor vehicle crashes are still a substantial cause of fatalities, and age waivers that allow younger teens to drive, may put them at increased risk for crashes and crash-related deaths. Gun-carrying and suicide also continue to be areas that require continued focus for injury prevention in teens. Primary care pediatricians are an important part of the multi-pronged approach for injury prevention for children of all ages. One study in this issue demonstrates a quality improvement program is feasible and effective in increasing injury prevention anticipatory guidance in pediatric practices.

For twenty-two years, the Injury Free Coalition for Kids® has been effective as a network of clinicians and injury prevention specialists working together with the goal to decrease preventable injuries in children and adolescents. This is done through the work in our hospitals and together with the communities we serve. The body of work in this issue is just a sample of the exemplary efforts by members in the Coalition to improve the lives of children through injury prevention. By applying the knowledge about the risks of injury along with implementing effective strategies for injury prevention, we strive for a world where preventable injuries are no longer a leading cause of death and disability in children.

